# Ginsenoside Rb1 prevents homocysteine-induced EPC dysfunction via VEGF/p38MAPK and SDF-1/CXCR4 activation

**DOI:** 10.1038/s41598-017-13436-7

**Published:** 2017-10-12

**Authors:** Tao-Hua Lan, Dan-Ping Xu, Man-Ting Huang, Ju-Xian Song, Huan-Lin Wu, Min Li

**Affiliations:** 1 Department of Cardiology, The Second Affiliated Hospital of Guangzhou University of Chinese Medicine, Guangzhou, P. R. China; 2 School of Chinese Medicine, Hong Kong Baptist University, Hong Kong, Hong Kong

## Abstract

Hyperhomocystinemia (HHcy) is known as an independent risk factor for cardiovascular disease. Our previous study showed that ginsenoside Rb1, the major active constituent of ginseng, prevents homocysteine (Hcy)-induced endothelial damage. However, the role of ginsenoside Rb1 in Hcy-induced dysfunction in endothelial progenitor cells (EPCs) remains unknown. In the study, we found that ginsenoside Rb1 reversed the Hcy-induced impairment of adhesive and migratory ability in EPCs which were significantly abolished by CXCR4 antagonist AMD3100 and VEGFR2 inhibitor SU5416. Ginsenoside Rb1 significantly reversed Hcy-induced SDF-1 reduction in the supernatant and in the serum. Ginsenoside Rb1 reversed downregulation of SDF-1 and VEGFR2 protein expression, inhibition of p38MAPK phosphorylation induced by Hcy. Re-endothelialization in balloon-injured carotid arteries significantly increased with EPCs transplant, and was even better with Rb1 treatment. This effect was significantly abolished by AMD3100. AMD3100 also decreased the number of CM-DiI labeled EPCs in injured arteries. Here we show for the first time that Rb1 prevents Hcy-induced EPC dysfunction via VEGF/p38MAPK and SDF-1/CXCR4 activation. These findings demonstrate a novel mechanism of the action of Rb1 that may have value in prevention of HHcy associated cardiovascular disease.

## Introduction

Hyperhomocystinemia (HHcy) has been recognized as an independent risk factor for atherosclerosis since 1969^1^. Even though low to moderately high levels of HHcy may not be atherogenic, HHcy may promote atherosclerosis in concert with other factors, such as mechanical injury^2^. It is widely accepted that endothelial dysfunction plays a crucial role in the pathogenesis and development of coronary atherosclerosis^3^. Endothelial dysfunction ultimately represents a negative balance between the magnitude of injury and the capacity for repair^4^. Endothelial progenitor cells (EPCs), derived primarily from the bone marrow, are precursors to mature endothelial cells with distinctive characteristics. EPCs circulate in the blood and contribute to injured vessel repair in response to vascular trauma or tissue ischemia^5^. Cytokine and vascular endothelial growth factors released at the site of injury promote EPCs from bone marrow to peripheral blood, which subsequently migrate to the injured site and differentiate into mature endothelial cells, leading to re-endothelialization and neovascularization^6^. Stromal cell-derived factor-1(SDF-1) enhances EPCs functions and SDF-1/CXCR-4 axis plays a crucial role in modulating the mobilization of EPCs from bone marrow^7^. It has been observed that homocystine (Hcy) reduces EPC numbers and impairs their functional capacity in patients with coronary artery disease (CAD)^8^, pulmonary arterial hypertension^9^, and stroke^10^. A reduction in the expression of vascular endothelial growth factor receptor-2 (VEGFR2) was shown in high Hcy level mice^11^ and VEGF/VEGFR inhibition was involved in Hcy-impaired angiogenesis^12^. It has been reported that p38 mitogen-activated protein kinase (p38MAPK) phosphorylation activation was involved in Hcy-induced proliferation reduction, especially in apoptosis caused cell death^13^. Activation of p38 MAPK phosphorylation regulates endothelial cell migration and is important in VEGF-induced angiogenesis^14^.

Ginseng, one of the most widely used herbal drugs, is becoming increasingly popular in Western countries for its alleged tonic effect and possible curative and restorative properties^15^. Ginsenosides are the major active constituents of ginseng. It has been reported that ginsenoside Rg1 promotes EPC migration and proliferation^16^, enhances EPCs’ angiogenic potency and prevents senescence of cells *in vitro* ^17^. Our previous study has showed that ginsenoside Rb1 via PI3K/Akt activation and PKC inhibition, prevents HUVECs from undergoing Hcy-induced endothelial dysfunction^18^. However, the role of ginsenoside Rb1 in Hcy-induced EPC dysfunction and its underlying mechanism remain unknown. In the current study, we hypothesize that VEGF/p38MAPK and SDF-1/CXCR4 activation be involved in the protective effect of ginsenoside Rb1 on Hcy-induced endothelial progenitor cell dysfunction.

## Results

### Ginsenoside Rb1 reversed Hcy-induced reduction of EPCs adhesion

**Figure 1:**
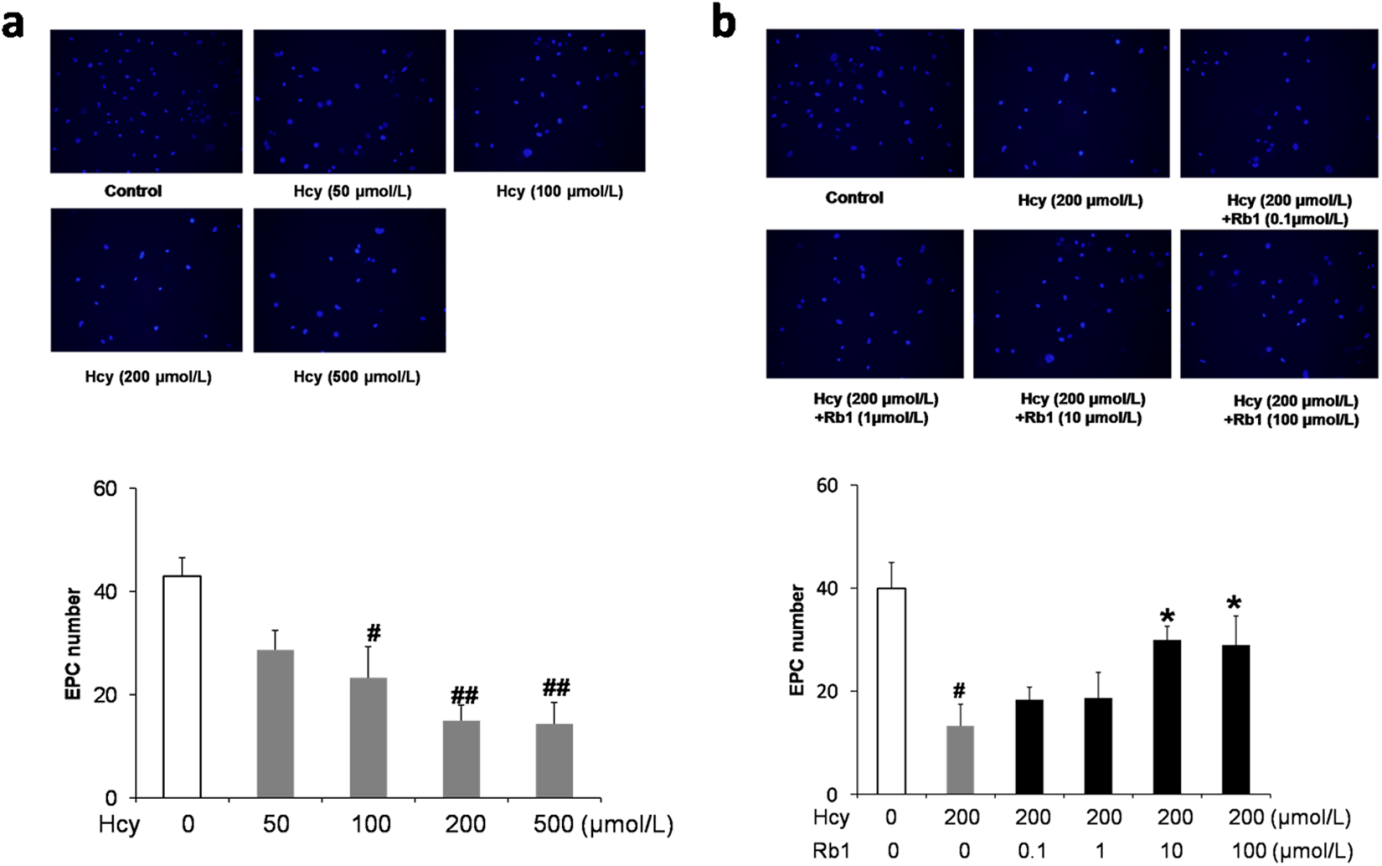
Ginsenoside Rb1 reversed Hcy-induced reduction of EPC adhesion ability to fibronetin. EPCs were treated with different concentration of Hcy (**a**), or various concentration of Rb1 with 200 μmol/L Hcy (**b**) for 48 h. 3 × 10^4^ EPCs were seeded into each well of a fibronectin-coated 24-well plate. After incubation at 37 °C for 30 min, cells were stained with DAPI for 10 min. Then the adherent EPCs were counted under the fluorescence microscope. Experiments were repeated three times and the data are shown as mean ± s.d. ^#^P < 0.05, ^##^P < 0.01 for Hcy-treated cells versus untreated control cells, *P < 0.05 for Rb1- treated cells versus 200 μmol/L Hcy-treated cells.

**Figure 2:**
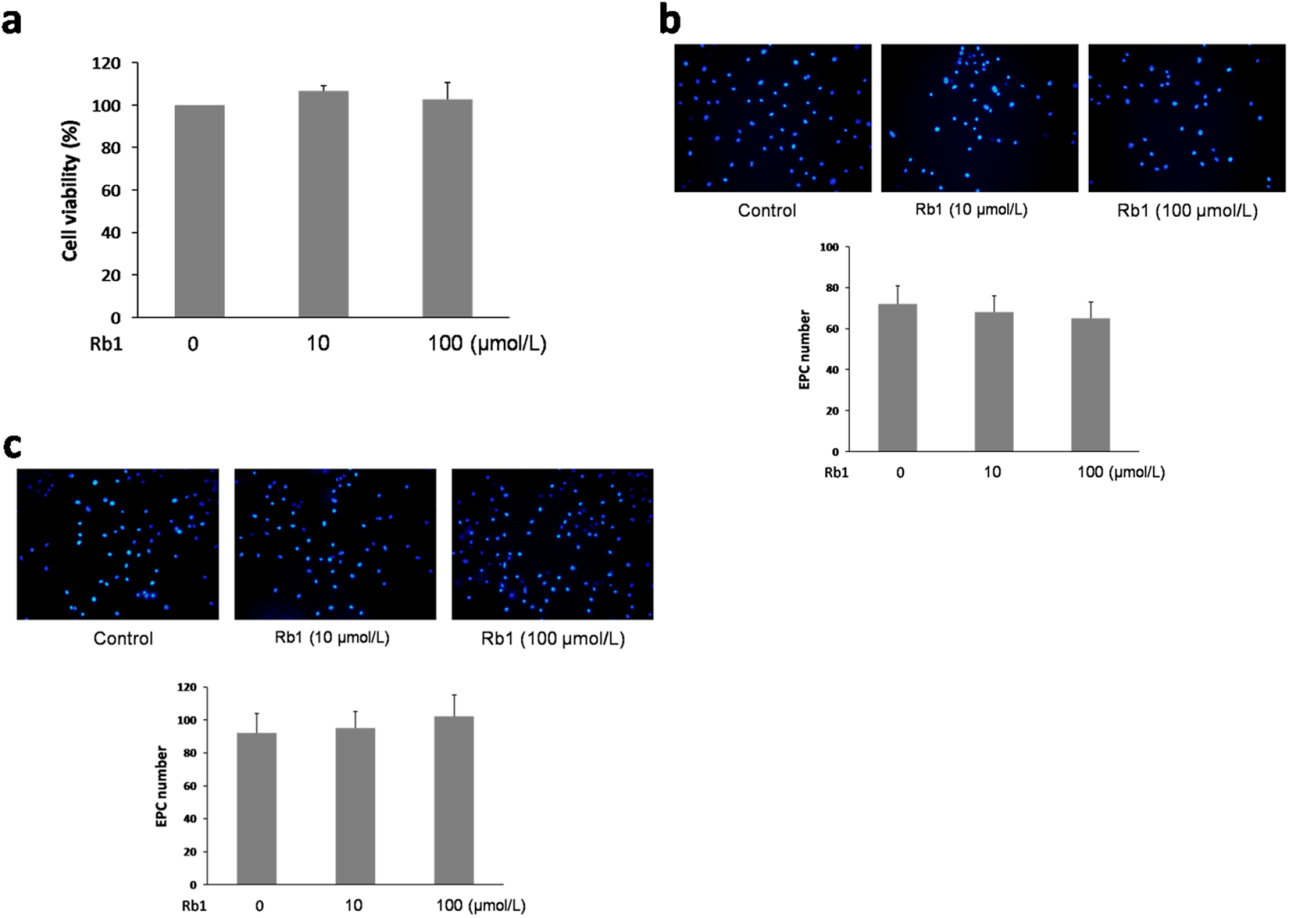
Ginsenoside Rb1 alone had no significant effects on EPC viability, adhesion and migration. (**a**) After treatment with indicated concentrations of Rb1 for 48 h, cell viability was evaluated by CCK-8 using a Microplate Reader. (**b**) After treatment with indicated concentrations of Rb1 for 48 h, 3 × 10^4^ EPCs were seeded into each well of a fibronectin-coated 24-well plate. After incubation at 37 °C for 30 min, cells were stained with DAPI for 10 min. Then the adherent EPCs were counted under the fluorescence microscope. (**b**) After treatment with indicated concentrations of Rb1 for 24 h, 3 × 10^4^ cells suspended in serum-free EGM-2 media with added drugs were placed in the upper chamber of a transwell insert. EGM-2 media with 20% FBS was added in the lower chamber. After incubation with drugs for another 24 h, the cells on the lower side of the filter were stained with DAPI for 10 min. Then the cells that had migrated to the lower filter were counted under the fluorescence microscope. Experiments were repeated three times and data are shown as mean ± s.d.

After stimulation with 50, 100, 200, or 500 μmol/L Hcy for 48 h, EPCs were seeded into each well of fibronectin-coated 24-well plate. The adherent EPCs were counted under the fluorescence microscope after incubation at 37 °C for 30 min. The number of EPCs adhered to fibronectin in the presence of Hcy showed a dose-dependent reduction (Fig. 1a). EPCs were also treated with indicated concentrations of ginsenoside Rb1 and 200 μmol/L Hcy for 48 h. As showed in Fig. 1b, Rb1 reversed the decreasing effect of Hcy on EPCs adhesion at concentrations of 10 and 100 μmol/L. Rb1 alone (either 10 or 100 μmol/L) has no significant effects on EPC viability and adhesion (Fig. 2a and b).

### Ginsenoside Rb1 reversed Hcy-induced reduction of EPCs migration

**Figure 3:**
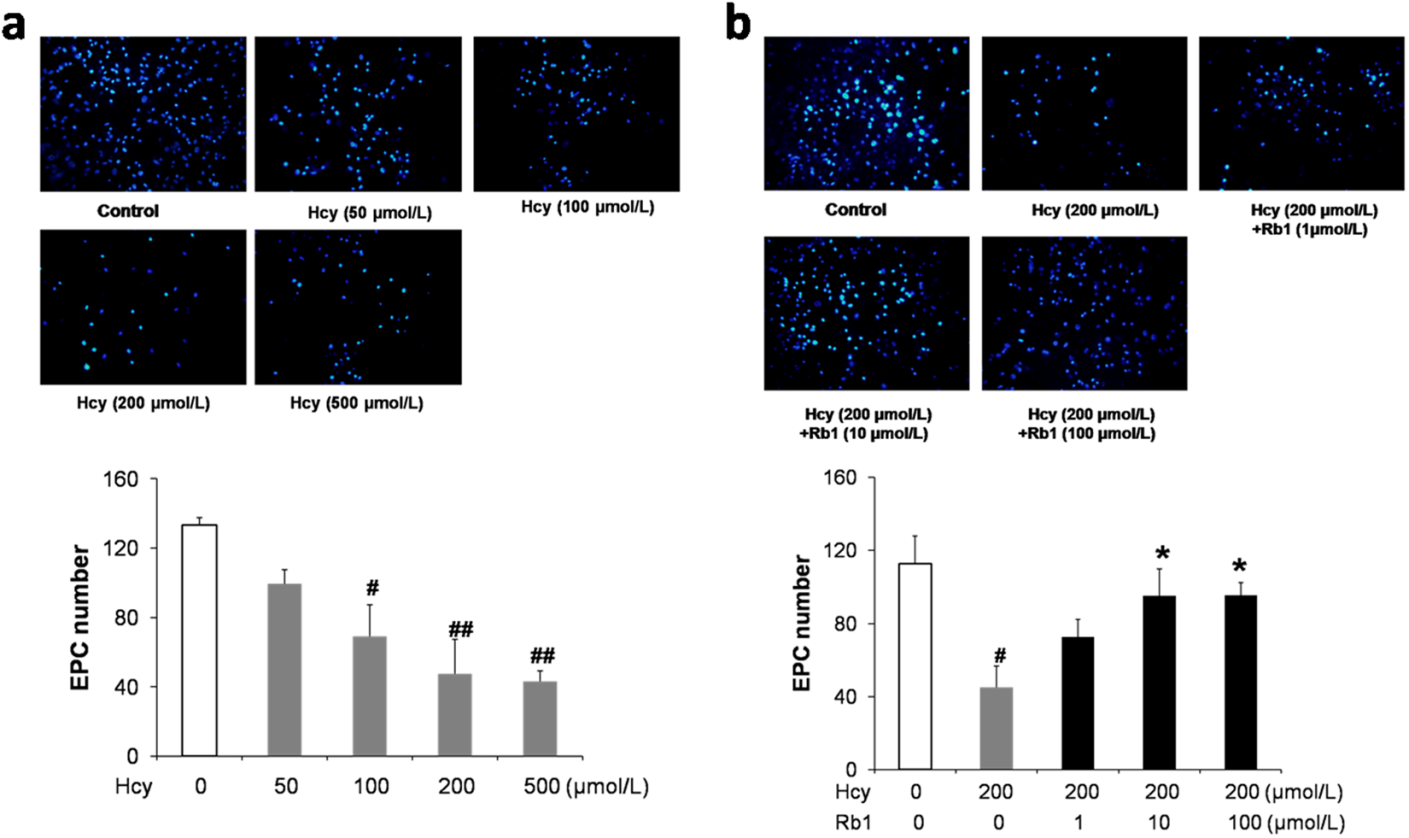
Ginsenoside Rb1 reversed Hcy-induced reduction of EPC migration. EPCs were treated with different concentrations of Hcy (**a**), or various concentrations of Rb1 and 200 μmol/L Hcy (**b**) for 24 h. Then 3 × 10^4^ cells suspended in serum-free EGM-2 media with added drugs were placed in the upper chamber of a transwell insert. EGM-2 media with 20% FBS was added in the lower chamber. After incubation with drugs for another 24 h, the cells on the lower side of the filter were stained with DAPI for 10 min. Then the cells that had migrated to the lower filter were counted under the fluorescence microscope. Experiments were repeated three times and data are shown as mean ± s.d. ^#^P < 0.05, ^##^P < 0.01 for Hcy-treated cells versus untreated control cells, *P < 0.05 for Rb1- treated cells versus 200 μmol/L Hcy-treated cells.

After stimulation with 50, 100, 200, or 500 μmol/L Hcy for 24 h, EPCs migration was measured by using the transwell chamber. EPC migration was inhibited by Hcy in a dose-dependent manner (Fig. 3a). EPCs were also treated with indicated concentrations of ginsenoside Rb1 and 200 μmol/L Hcy for 24 h before performing transwell migration assay. As showed in Fig. 2b, Rb1 reversed the decreasing effect of Hcy on EPCs migration at concentrations of 10 and 100 μmol/L. Rb1 alone (either 10 or 100 μmol/L) has no significant effects on EPC migration (Fig. 2c).

### CXCR4 antagonist and VEGFR2 inhibitor abolished ginsenoside Rb1-induced improvement on EPCs adhesion and migration

**Figure 4:**
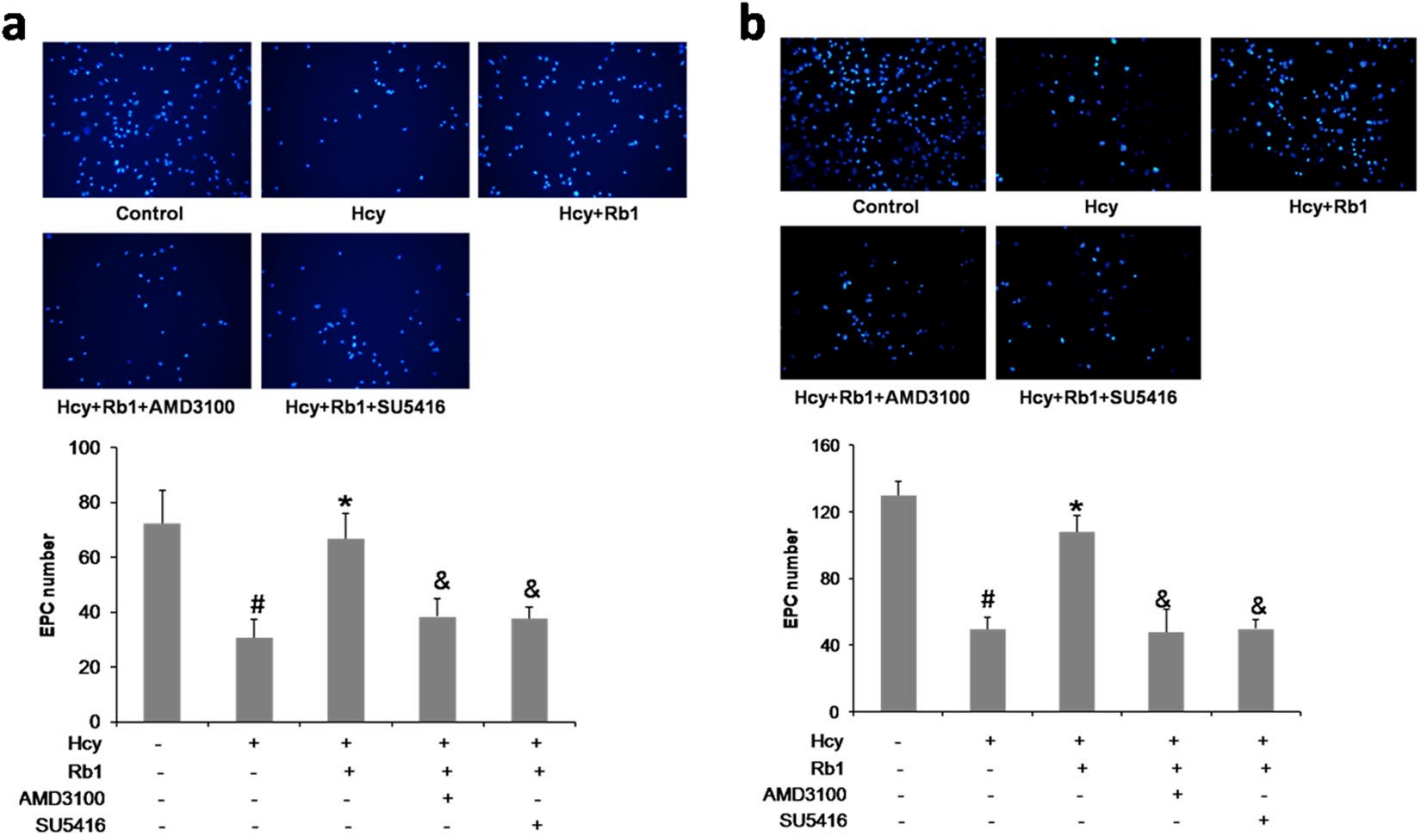
CXCR4 antagonist and VEGFR2 inhibitor abolished ginsenoside Rb1-induced improvement on EPCs adhesion and migration. EPCs were treated with 200 μmol/L Hcy and/or 10 μmol/L Rb1, or pretreated with 100 ng/ml CXCR4 antagonist AMD3100 or 10 μmol/L VEGFR2 inhibitor SU5416 for 48 h. Cell adhesion (**a**) and migration (**b**) ability were tested. Experiments were repeated three times and data are shown as mean ± s.d. ^#^P < 0.05 for Hcy-treated cells versus untreated control cells, *P < 0.05 for Rb1- treated cells versus 200 μmol/L Hcy-treated cells, ^&^P < 0.05 compare with Rb1- treated cells.

EPCs were treated with 200 μmol/L Hcy and/or 10 μmol/L Rb1 for 48 h, or pretreated with 100 ng/ml AMD3100 (CXCR4 antagonist) or 10 μmol/L SU5416 (VEGFR2 inhibitor) for 1 h. Cell adhesion and migration ability were tested. As showed in Fig. 4, CXCR4 antagonist and VEGFR2 inhibitor abolished ginsenoside Rb1-induced improvement on EPCs adhesion and migration. These results indicate that SDF-1 and VEGF may be involved in the Rb1-induced increase of EPCs adhesion and migration.

### Ginsenoside Rb1 reversed Hcy-induced SDF-1 reduction in EPCs

**Figure 5:**
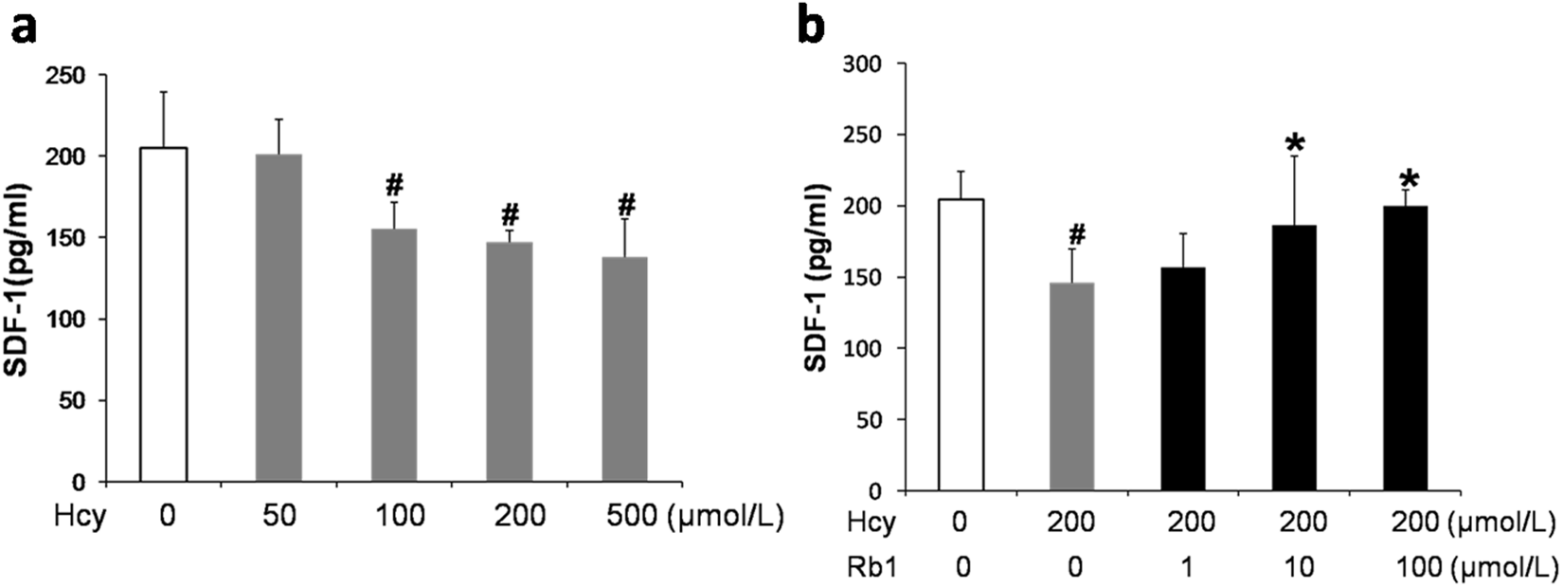
Ginsenoside Rb1 reversed Hcy-induced SDF-1 reduction in EPCs. After treatment with indicated concentrations of Hcy (**a**), or indicated concentrations of Rb1 with 200 μmol/L Hcy (**b**) for 48 h, the supernatants were collected to evaluate SDF-1 levels by ELISA. Data are shown as mean ± s.d (n = 3). ^#^P < 0.05 for Hcy-treated cells versus untreated control cells, *P < 0.05 for Rb1- treated cells versus 200 μmol/L Hcy-treated cells.

After treatment with indicated concentrations of Hcy and ginsenoside Rb1 for 48 h, the supernatant were collected to evaluate SDF-1 level by ELISA. As showed in Fig. 5, Hcy significantly reduced SDF-1 in a dose-dependent manner, while Rb1 significantly reversed Hcy induced SDF-1 reduction at concentrations 10 μmol/L.

### Ginsenoside Rb1 upregulated SDF-1 and VEGFR2 in EPCs

**Figure 6:**
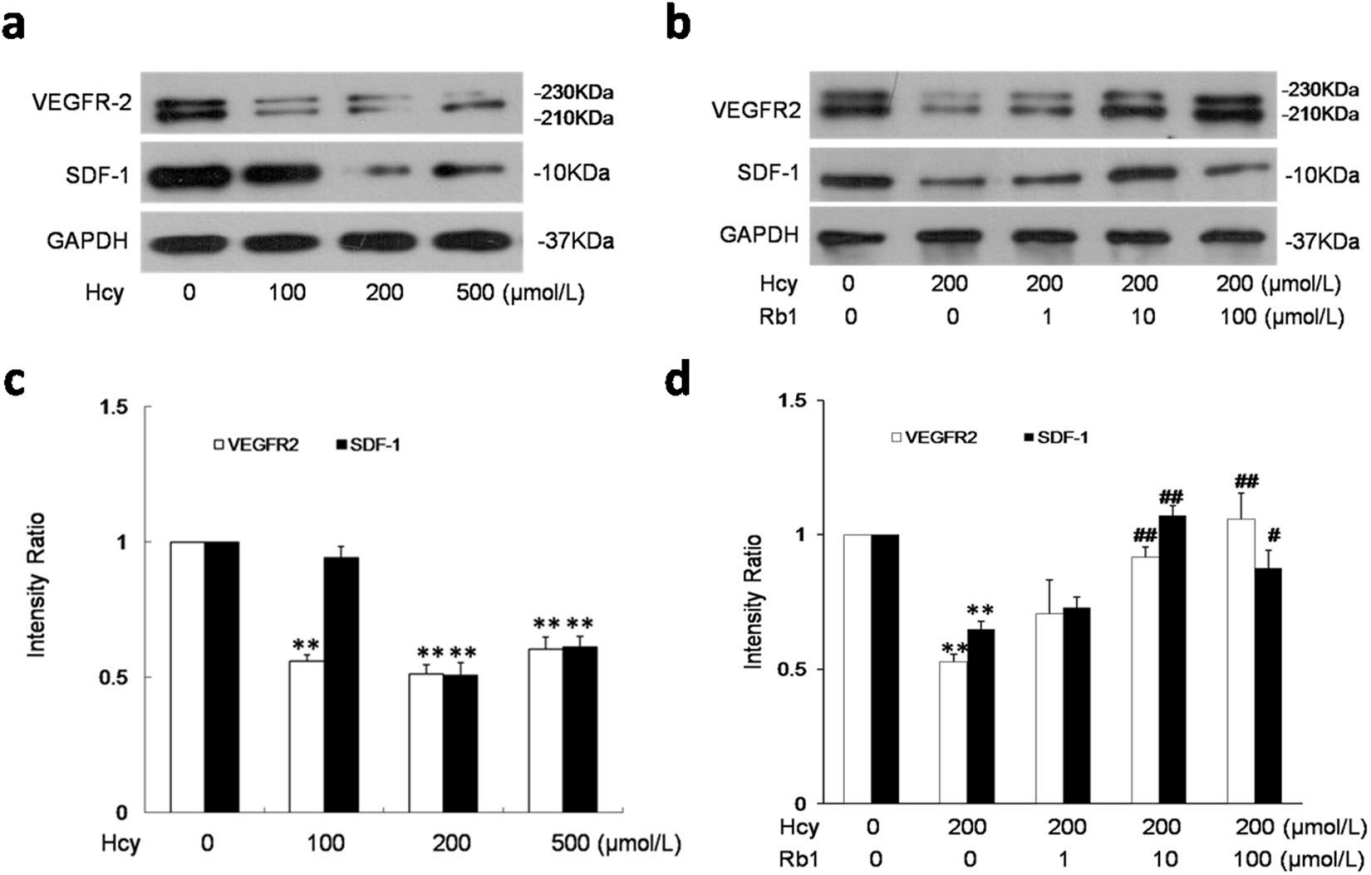
Ginsenoside Rb1 reversed Hcy-induced down-regulation of SDF-1 and VEGFR2 in EPCs. EPCs were treated with indicated concentrations of Hcy (**a**), or indicated concentrations of Rb1 with 200 μmol/L Hcy (**b**) for 48 h, and the expression of SDF-1 and VEGFR2 were determined by Western blot analysis. VEGFR-2/ GAPDH ratio and SDF-1/GAPDH ratio were quantified as shown in (**c**) and (**d**). White space was used to make explicit for the grouping of blots cropped from different parts of the same gel or from different gels. Experiments were repeated three times and data are shown as mean ± s.d. **P < 0.01 for Hcy-treated cells versus untreated control cells. ^#^p < 0.05, ^##^p < 0.05 for Rb1-treated cells versus 200 μmol/L Hcy-treated cells.

To examine involvement of SDF-1 and VEGFR2 in the effect of Hcy and Rb1 on EPCs adhesion and migration, the expression of SDF-1 and VEGFR2 were tested by Western blotting. As showed in Fig. 6, Hcy resulted in a significant downregulation of SDF-1 and VEGFR2. Rb1 reversed the downregulation of SDF-1 and VEGFR2 induced by Hcy. These results indicate that the upregulation of SDF-1 and VEGFR2 were involved in the Rb1-induced increase of EPC adhesion and migration.

### Ginsenoside Rb1 activated p38MAPK phosphorylation in EPCs

**Figure 7:**
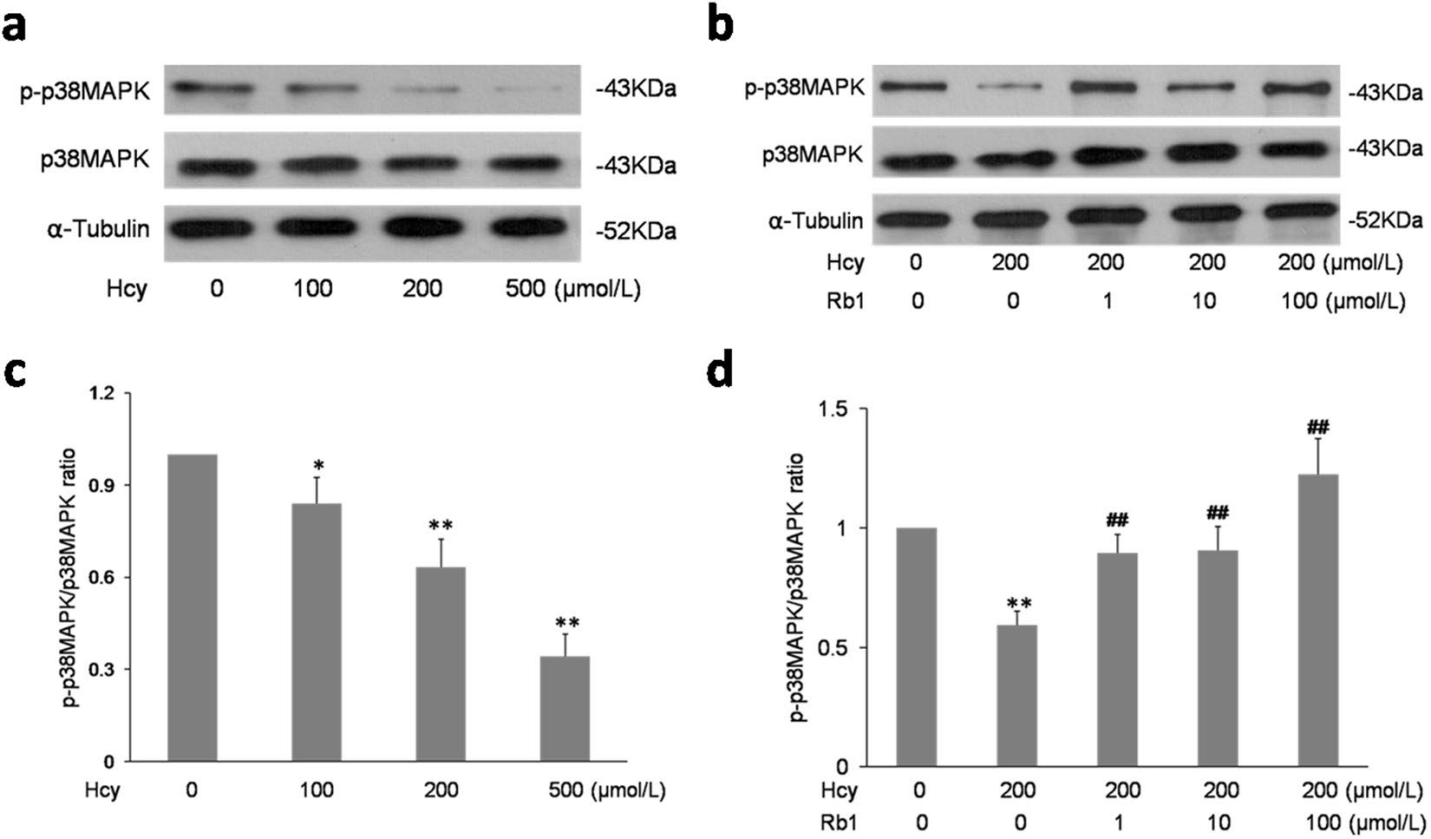
Ginsenoside Rb1 reversed Hcy-induced dephosphorylation of p38MAPK in EPCs. EPCs were treated with indicated concentrations of Hcy (**a**), or indicated concentrations of Rb1 with 200 μmol/L Hcy (**b**) for 48 h. The expression of p38MAPK and p-p38MAPK were determined by Western blot analysis. p-p38MAPK/38MAPK ratio were quantified as shown in (**c**) and (**d**). White space was used to make explicit for the grouping of blots cropped from different parts of the same gel or from different gels. Experiments were repeated three times and data are shown as mean ± s.d. *P < 0.05, **P < 0.01 for Hcy-treated cells versus untreated control cells. ^#^p < 0.05 for Rb1-treated cells versus 200 μmol/L Hcy-treated cells.

To examine involvement of p38MAPK in the effect of Hcy and Rb1 on EPCs adhesion and migration, p38MAPK phosphorylation and total p38MAPK were tested by Western blotting. As showed in Fig. 7, Hcy inhibited p38MAPK phosphorylation in a dose-dependent manner. However, Hcy had no effect on total p38MAPK. Rb1 reversed Hcy-induced inhibition of p38MAPK phosphorylation without altering the protein expression of total p38MAPK. These results indicate that the p38MAPK phosphorylation may be involved in the Rb1-induced increase of EPCs adhesion and migration.

### Ginsenoside Rb1 promoted re-endothelialization in injured carotid artery

**Figure 8:**
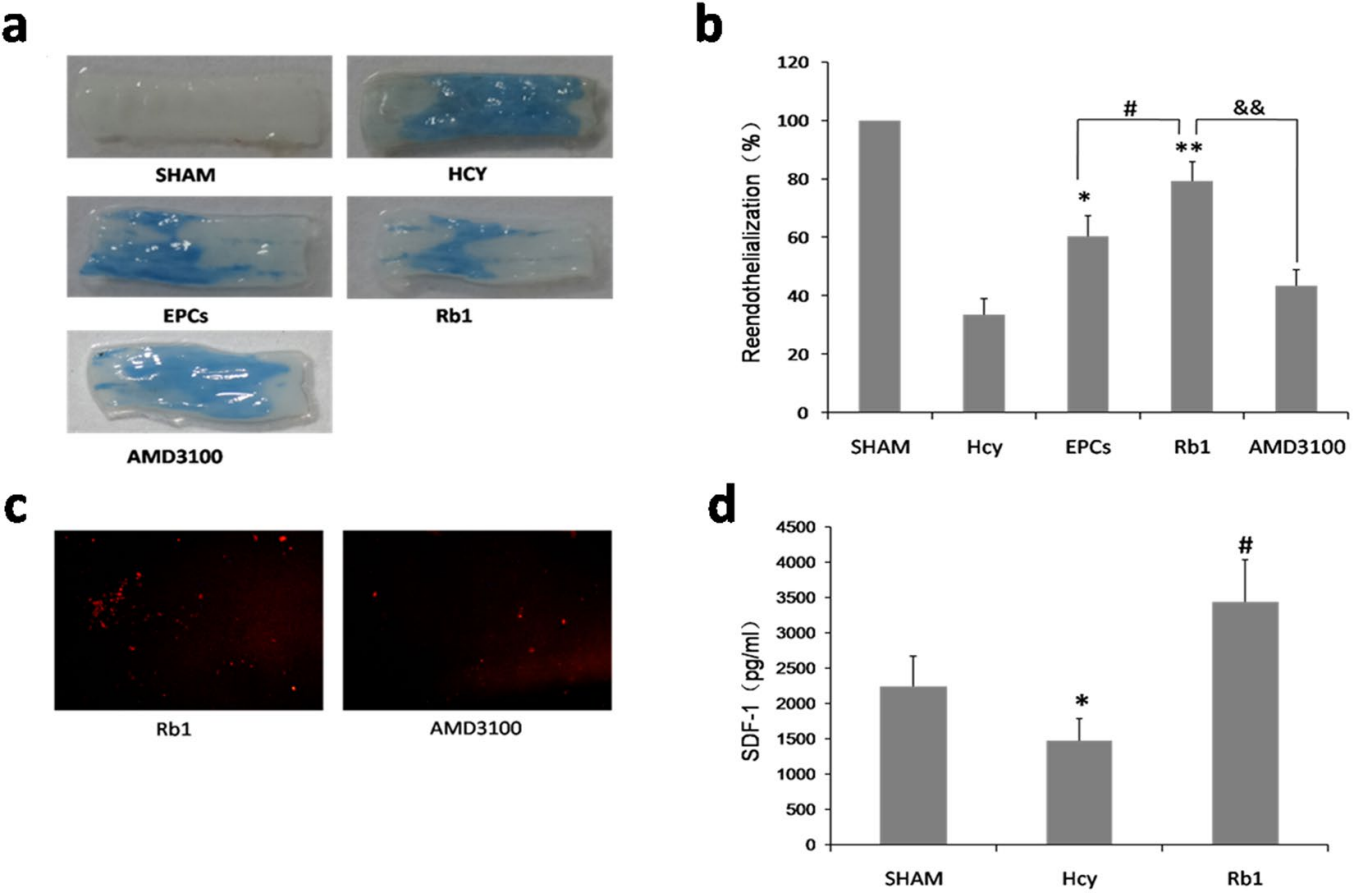
Ginsenoside Rb1 promoted EPCs homing in on injured carotid arteries. (n = 8) The carotid artery balloon injury model was established after 4 weeks of intragastric administration with L-methionine in rats. (**a**,**b**) EPCs with or without CXCR4 antagonist AMD3100 pretreatment were injected through tail vein. Reendothelialization in injured arteries were observed by Evans blue after 2 weeks of intraperitoneal injection of 10 mg/kg ginsenoside Rb1. (**c**) CM-DiI labeled EPCs with or without CXCR4 antagonist AMD3100 pretreatment were injected through tail vein. The number of CM-DiI labeled EPCs in injured arteries was counted with a fluorescence microscope after 2 weeks intraperitoneal injection of 10 mg/kg ginsenoside Rb1. (**d**) Serum was collected to evaluate SDF-1 level by ELISA.

The carotid artery balloon injury model was established after 4 weeks of intragastric administration with L-methionine in rats. EPCs with or without CXCR4 antagonist AMD3100 pretreatment were injected via tail vein. To evaluate the effect of Rb1 on re-endothelialization, the carotid endothelial recovery was observed by Evans blue staining after 2 weeks intraperitoneal injection of 10 mg/kg ginsenoside Rb1. Non-endothelialized lesions were marked by blue staining, whereas the reendothelialized area appeared white (Fig. 8a). The re-endothelialization area in the EPC-transplanted rats and Rb1 treated rats were significantly larger than that in Hcy rats, and Rb1 treatment has better effects than EPCs only (Fig. 8a and b). The re-endothelialization area in AMD3100 treated rats was significantly smaller than that in Rb1 treated rats. These results indicate that Rb1 treatment promoted re-endothelialization in injured carotid arteries and that the effect was abolished by CXCR4 antagonist.

### SDF-1/CXCR4 axis is involved in ginsenoside Rb1-promoted EPCs homing in on injured carotid artery

The carotid artery balloon injury model was established after 4 weeks of intragastric administration with L-methionine in rats. CM-DiI labeled EPCs with or without CXCR4 antagonist AMD3100 pretreatment were injected via tail vein. The numbers of CM-DiI- labeled EPCs in injured arteries were counted under a fluorescence microscope after 2 weeks intraperitoneal injection of 10 mg/kg ginsenoside Rb1. As showed in Fig. 8c, the number of CM-DiI- labeled EPCs in injured arteries of AMD3100 treated rats was significantly less than that of Rb1 rats. These results indicate that Rb1 treatment promoted EPCs homing finding injured carotid arteries and that the effect is related to SDF-1/CXCR4 axis.

### Ginsenoside Rb1 reversed Hcy-induced SDF-1 reduction in serum

Two weeks after the surgery, serum was collected to evaluate SDF-1 level by ELISA. As showed in Fig. 8d, the SDF-1 level in Hcy rats was significantly lower than that in sham rats, while the SDF-1 level in Rb1-treated rats was significantly higher than in Hcy rats. These results indicate that Hcy reduced the secretion of SDF-1 which was reversed by Rb1 treatment, leading to promoting EPCs homing in on injured arteries.

## Discussion

In the study, we show for the first time that Rb1 prevents Hcy-induced EPC dysfunction via VEGF/p38MAPK and SDF-1/CXCR4 activation. We found that ginsenoside Rb1 reversed Hcy-induced impairment of adhesive and migratory ability in EPCs and significantly reversed Hcy-induced SDF-1 reduction in the supernatant and in the serum. Ginsenoside Rb1 reversed downregulation of SDF-1 and VEGFR2 protein expression, inhibition of p38MAPK phosphorylation induced by Hcy. Re-endothelialization in balloon-injured carotid arteries significantly increased with EPCs transplant, and was even better with Rb1 treatment, which significantly abolished by CXCR4 antagonist AMD3100.

The vascular endothelium is a key player in vascular homeostasis. Endothelial dysfunction and injury are considered to be the first steps in development and progression of atherosclerosis^19^. Endothelial progenitor cells (EPCs), which were reported for the first time in peripheral circulation in 1997^20^, contribute substantially to preservation of a structurally and functionally intact endothelium. EPCs home in on to the sites of endothelial injury and ischemia, where they proliferate, differentiate and integrate into the endothelial layer or exert a paracrine function by producing vascular growth factors. Cell-based therapy to repair vascular integrity is being evaluated as a strategy for treating cardiovascular diseases with excess morbidity and mortality. Bone marrow-derived EPCs, as a potential new strategy in regenerative medicine, are already being used therapeutically in several diseases including acute myocardial infarction, chronic myocardial infarction, dilated cardiomyopathy and peripheral arterial occlusive disease^21^.

In patients with cardiovascular risk factors, EPC numbers in circulation are decreased and their function is impaired; this ultimately leads to progressive endothelial dysfunction^22^. Hyperhomocystinemia (HHcy) is accepted as a common risk factor for cardiovascular disease^23^. The numbers of circulating and differentiated EPCs are both inversely correlated with Hcy^8^. Hcy accelerates the onset of EPC senescence, leading to cellular dysfunction^24^. In this study, we confirmed that Hcy induced impairment of adhesive and migratory ability in EPCs without causing EPCs death within the concentration of 500 μmol/L for 48 h (data not shown).

Ginseng is used world-wide for its alleged tonic effects and possible curative and restorative properties. Growing evidence shows that ginsenoside Rb1, the most active ingredient of ginseng, has multiple pharmacological effects on the cardiovascular system by reducing oxidative stress^25^, activating eNOS activity and inducing NO release^26,27^. Our previous study has showed that ginsenoside Rb1 prevents HUVECs from Hcy-induced endothelial dysfunction via PI3K/Akt activation and PKC inhibition^18^. However, the role of ginsenoside Rb1 on Hcy-induced dysfunction in EPCs remains unknown. In this study, we found that ginsenoside Rb1 reversed the Hcy-induced impairment of EPCs’ adhesive and migratory abilities. *In vivo*, Rb1 promoted EPCs’ homing in on injured arteries leading to re-endothelialization.

Vascular endothelial growth factor (VEGF) exerts its effects via binding to specific receptors and initiates a cascade of cellular protein phosphorylations by protein kinases, including p38 MAPK^28^, PI3K/Akt^29^ and ERK1/2^30^. Endothelial cell migration, a key step in angiogenesis, is regulated spatiotemporally by multiple pathways including MAPKs^14^. Activation of MAPKs is important in VEGF-induced angiogenesis, which is highly involved with p38. Our study found that Hcy reduced the expression of VEGFR2 and the phosphorylation of p38MAPK in EPCs which were significantly reversed by Rb1. The Rb1-induced improvements of adhesive and migratory ability in EPCs were abolished by VEGFR2 inhibitor SU5416. The data implied that VEGFR2/ p38MAPK activation was involved in the effects of ginsenoside Rb1 on improving functions of EPCs.

Stromal cell-derived factor-1 (SDF-1) is a G protein-coupled receptor and inducible chemokine that regulates multiple physiological processes. Induction of SDF-1 expression plays a major role in re-endothelialization of injured vessels^31^ and revascularization of ischemic tissues^32^ while appears to increase in states of endothelial dysfunction and vascular injury. It is accepted that the SDF-1/CXCR4 axis (which is inhibited by AMD3100) exerts a crucial role in modulating the mobilization of proangiogenic hematopoietic cells from bone marrow. In the present study, we found that Hcy reduced SDF-1 level both in the supernatant of EPCs and in the serum of carotid artery balloon injuried rat. The protein expression of SDF-1 in EPCs also decreased in the presence of Hcy. The data indicated that Hcy-induced EPCs dysfunction is partially due to the reduction of SDF-1. It was observed in this study that ginsenoside Rb1 significantly reversed the reduction of SDF-1 caused by Hcy and promoted the re-endothelialization in injured arteries, which were abolished by CXCR4 antagonist AMD3100. The findings imply that SDF-1/CXCR4 axis is involved in the process by which ginsenoside Rb1 enhances EPC functions.

The pharmacokinetics of ginsenoside Rb1 by LC-MS/MS method has been reported in recent years. After oral administration (50 mg/kg) and intravenous administration (10 mg/kg) of Rb1 in rats^33^, the main pharmacokinetic parameters showed that for oral administration, Rb1 reached the peak in 2.0 h with concentration of 6.1 mg/l and AUC0-∞ 66.8 h mg/l. For intravenous administration, Rb1 reached the peak in 0.083 h with concentration of 199.6 mg/l and AUC0-∞ 1859.1 h mg/l. Another pharmacokinetic study of Rb1 with intravenous administration (10 mg/kg) showed that Rb1 reached the peak immediately with concentration of 100000 ng/ml and AUC0-∞ 932.2 h mg/l^34^. Due to the poor bioavailability of oral administration, intravenous or intraperitoneal injection would be the proper way for Rb1 administration.

In conclusion, our present study indicates that ginsenoside Rb1 prevents homocystine-induced dysfunction in endothelial progenitor cells via VEGF/ p38MAPK and SDF-1/CXCR4 activation. Although the signaling pathways need to be defined in more detail, our results demonstrate a novel mechanism of the effects of Rb1 on HHcy–induced endothelial dysfunction in EPCs and provide new evidence for the potential clinical application of ginseng in prevention of HHcy associated cardiovascular disease.

## Materials and Methods

### Reagents and antibodies

Ginsenoside Rb1(Rb1) purchased as a reference compound (purity > 98%) from the Division of Chinese Materia Medica and Natural Products, National Institute for the Control of Pharmaceutical and Biological Products (NICPBP), Ministry of Public Health, China. EGM-2MV was purchased from Lonza (Switzerland). DiI-ac-LDL was purchased from Molecular Probe (Thermo Scientific, Waltham, MA, USA). Antibodies against Flk-1, CD133 and CD34 were purchased from Bioss (Beijing, China). Trypsin, penicillin, streptomycin, CM-DiI and FBS were purchased from Gibco (Invitrogen, Carlsbad, CA, USA). Fibronetin was purchased from BD Biosciences (San Jose, CA, USA). L-methionine, SST, FITC-UEA, Hcy, Ficoll, H2DCF-DA, DAPI, Evans blue, AMD3100, SU5416 and GAPDH antibody were purchased from Sigma (St Louis, MO, USA). SDF-1 ELISA Kit was purchased from Cusabio (Wuhan, China). Antibodies against p38MAPK, phosphor-p38MAPK, SDF-1, VEGFR2 and tubulin were purchased from Cell Signaling Technology (Boston, MASS, USA). Fogarty catheter was purchased from Edwards Lifesciences (Irvine, CA, USA).

### Bone marrow-derived EPCs culture

Male Sprague-Dawley rats (specific-pathogen-free, weighing 180 to 200 g) were provided by the Experimental Animal Center of Guangzhou University of Chinese Medicine. The animal experiment was approved by the Animal Care and Use Committee of Guangzhou University of Chinese Medicine. All animal care and procedures conform to the Council for International Organizations of Medical Sciences (CIOMS) guidelines. Marrow harvested from the femur and tibia of SD rats, then individual cells were suspended and added to Ficoll at a ratio of 1:1. The mixture was then centrifuged at 2,000 rpm for 20 min at 20 °C to separate the cells into three layers. The middle layer, which was white and cloudy and consisted of the mononuclear cells, was gently removed and transferred to a new centrifuge tube. The cells were washed twice with PBS, seeded in fibronectin-coated T25 culture flask in EGM-2MV (Endothelial cell basal medium-2, plus VEGF, R3-IGF-1, rhEGF, rhFGF-B, GA-1000, hydrocortisone and ascorbic acid) supplemented with 20% FBS. Cultures were maintained at 37 °C in humidified 5% CO_2_ incubator. Three days later, non-adherent cells were washed off with PBS, and fresh media was added to the cultures every 3 days. EPCs were identified by their typical appearance, DiI-ac-LDL uptake, FITC-UEA binding and the presence of Flk-1, CD133, CD34. EPCs were divided into the following groups: control, Hcy groups (stimulated with 50, 100, 200, or 500 μmol/L Hcy), ginsenoside Rb1 groups (0.1, 1, 10 and 100 μmol/L Rb1 with 200 μmol/L Hcy). EPCs were pretreated with CXCR4 antagonist AMD3100 (100 ng/ml) or VEGFR2 inhibitor SU5416 (10 μmol/L) for 1 h before being treated with 10 μmol/L Rb1 and 200 μmol/L Hcy for adhesion and migration assay.

### EPCs viability assay

Cell viability was monitored by the CCK-8 assay. Briefly, EPCs were plated at a density of 1 × 10^4^ cells/well in a 96-well plate and routinely incubated for 48 h. After treatment, viable cells were stained with CCK-8 (100 μl/well, 2 h). Absorbance was measured at 450 nm by a microplate reader (Thermo, Boston, MA, USA). Results were expressed as percentages of control group.

### EPCs adhesion assay

3 × 10^4^ EPCs per well were seeded on fibronectin-coated 24-well plates after being treated with indicated concentrations of Hcy and ginsenoside Rb1 for 48 hours, or pretreated with AMD3100 or SU5416 for 1 h, then were cultured for 30 min in media with indicated drugs. Plates were washed three times with PBS, and incubated with 0.5 mg/L of DAPI for 10 min at 37 °C in the dark. Adherent cells were counted under a fluorescent microscope (magnified 200×).

### EPCs migration assay

After being treated with indicated concentrations of Hcy and ginsenoside Rb1 for 48 h, or pretreated with AMD3100 or SU5416 for 1 h, 2 × 10^4^ EPCs were resuspended in serum-free EBM-2 with indicated drugs and then were seeded in the upper chamber (pore size 8 µm) of transwell inserts. EBM-2 containing 20% FBS was added to the lower chamber. The transwell migration assay was performed at 37 °C for 24 hours. After removal of the remaining cells on the top side of filters using cotton swabs, the filters were incubated with 0.5 mg/L of DAPI for 10 min at 37 °C in the dark. The cells on the bottom side were washed three times with PBS and were counted under a fluorescent microscope (magnified 200×).

### Measurement of SDF-1 level in supernatant

EPCs were treated with indicated concentrations of Hcy and ginsenoside Rb1 for 48 hours. Then the supernatants were collected. The SDF-1 level in the supernatants was evaluated by ELISA following the manufacturer’s protocol.

### Western blot analysis

EPCs were lysed after treatment with indicated concentrations of Hcy and ginsenoside Rb1. Whole cell extract (40 μg) from each sample was analyzed with specific antibodies against p38MAPK (1:1000 dilution), phosphor-p38MAPK (1:1000 dilution), SDF-1 (1:1000 dilution) and VEGFR2 (1:1000 dilution). Incubation with monoclonal GAPDH (1:10000 dilution) or tubulin (1:1000 dilution) was performed as the loading control. The blots were detected on Kodak X-Omat film by the enhanced chemiluminescence. Quantification of band intensity was performed using the ImageJ software.

### The carotid artery balloon injury rat model

Male Sprague-Dawley rats (specific-pathogen-free, weighing 250 to 300 g) were obtained from the Experimental Animal Center of Guangdong Province. All rats were kept in a room maintained at 24 °C with a 12-h light/dark cycle and fed standard rat chow. The animal experiment was approved by the Animal Care and Use Committee of Guangzhou University of Chinese medicine. All animal care and procedures conform to the Council for International Organizations of Medical Sciences (CIOMS) guidelines. Rats were randomized into Sham group, Hcy group, EPCs group, Rb1 group and AMD3100 group (n = 8). Rats in Hcy group EPCs group, Rb1 group and AMD3100 group were administrated L-methionine via oral gavage at a rate of 1 g·kg^−1^·day^−1^ for 4 weeks. Succinylsulfathiazole (SST) (0.5 g·kg^−1^·day^−1^) was added to the drinking water of rats in order to avoid bacterial proliferation and subsequent folate production. After 4 weeks, rats were anesthetized with 10% chloral hydrate (350 mg/kg intraperitoneally). The left common carotid arteries were injured with a Fogarty 2 F catheter. Briefly, the catheter was inserted through the external carotid artery and introduced into the common carotid artery, then inflated with 300 μL saline. The inner surface of the common carotid artery was rubbed back and forth three times. The catheter was then removed and the external carotid artery was ligated. Rats in sham group underwent the same operation, except that the balloon was not inserted. All animals were sent back to the SPF animal experimental center of Guangdong Provincial Hospital of Chinese Medicine after intramuscular injection of Certriaxone Sodium (0.1 g/kg). Rats in the EPCs group and Rb1 group received i.v. injections of EPCs, while rats in the AMD3100 group were received i.v. injections of 100 ng/ml AMD3100 pretreated EPCs within 24 h. Rats in the Rb1 group and AMD3100 group were also intraperitoneally injected with 10 mg/kg ginsenoside Rb1 per day for 2 weeks.

### Analysis of Re-endothelialization of Arterial Segments

14 days after surgery, rats were injected with 2% Evans blue (40 mg/kg) via tail vein 10 min before sacrifice, followed by perfusion with 4% paraformaldehyde in saline for 5 min. The left common carotid arteries (1 cm) were collected from the carotid bifurcation, incised longitudinally, and photographed under a dissecting microscope. Re-endothelialized areas were defined macroscopically as those areas not stained by the Evans blue dye and quantified with Image-Pro Plus software version 6.0.

### CM-DiI labeled EPCs counting

Male Sprague-Dawley rats were subjected to balloon injury of the left common carotid artery after 4 weeks intragastric administration with L-methionine. Before intravenous injection, EPCs with or without 100 ng/ml AMD3100 pretreatment were incubated in 4 μg/ml CM-DiI for 5 min at 37 °C, and then for an additional 15 min at 4 °C. Rats received i.v. injections of CM-DiI labeled EPCs, followed by intraperitoneally injected with 10 mg/kg ginsenoside Rb1 per day for 2 weeks. 14 days after surgery, rats were sacrificed and the left common carotid arteries (1 cm) were collected, incised longitudinally. The numbers of CM-DiI labeled EPCs in injured arteries were counted under a fluorescence microscope (magnified 100×).

### Measurement of SDF-1 level in serum

Rats were fasted overnight and anesthetized with 10% chloral hydrate (350 mg/kg, i.p.). Blood was collected the next morning and centrifuged at 4000 rmp for 10 min at 4 °C after 2 h at room temperature. Serum was separated and stored at −80 °C for assay. The serum SDF-1 levels were measured by ELISA.

### Statistical analysis

All *in vitro* data were from at least three independent experiments. Results were expressed as the mean ± SD. Statistical comparison among multiple groups was performed by one-way ANOVA followed by LSD test using the SPSS 13.0 software (SPSS Inc, Chicago, Ill). A value of p < 0.05 was considered statistically significant.
